# Supplementary material for: Comparative multiomics analysis of cell physiological state after culture in a basket bioreactor
Source: Sci Rep. 2022 Nov 23;12:20161. doi: 10.1038/s41598-022-24687-4 (PMC9686226; doi:10.1038/s41598-022-24687-4)
Supplement: Supplementary file 1 — Supplementary Information 1. [file 41598_2022_24687_MOESM1_ESM.zip › raw data/Transcriptomic raw data/6.Enrichment/1.GO/TE1vsTC1/GSEA/TE1/PROTEIN_CATABOLIC_PROCESS/PROTEIN_CATABOLIC_PROCESS(GO_0030163).html]

Details for gene set PROTEIN\_CATABOLIC\_PROCESS(GO:0030163)[GSEA]


|  || Dataset | readcount.sample |
| Phenotype | sample.cls |
| Upregulated in class | TE1 |
| GeneSet | PROTEIN\_CATABOLIC\_PROCESS(GO:0030163) |
| Enrichment Score (ES) | 0.42567128 |
| Normalized Enrichment Score (NES) | 1.8240119 |
| Nominal p-value | 0.0 |
| FDR q-value | 0.19523181 |
| FWER p-Value | 0.494 |
Table: GSEA Results Summary

  

Fig 1: Enrichment plot: PROTEIN\_CATABOLIC\_PROCESS(GO:0030163)
  
Profile of the Running ES Score & Positions of GeneSet Members on the Rank Ordered List

  

| PROBE | DESCRIPTION   (from dataset) | GENE SYMBOL | GENE\_TITLE | RANK IN GENE LIST | RANK METRIC SCORE | RUNNING ES | CORE ENRICHMENT || 1 | 103218706 | LOC103218706 |  |  | 541 | Infinity | -0.0241 | Yes |
| 2 | 103214879 | LOC103214879 |  |  | 1075 | 3.386 | 0.0043 | Yes |
| 3 | 103222611 | PSMA8 |  |  | 1086 | 3.333 | 0.0553 | Yes |
| 4 | 103241474 | SIAH2 |  |  | 2473 | 1.868 | 0.0221 | Yes |
| 5 | 119618125 | UCHL3 |  |  | 3318 | 1.641 | 0.0096 | Yes |
| 6 | 103216824 | ATE1 |  |  | 3684 | 1.577 | 0.0176 | Yes |
| 7 | 103217971 | CUL3 |  |  | 3820 | 1.553 | 0.0355 | Yes |
| 8 | 103226162 | PSMA2 |  |  | 3924 | 1.537 | 0.0546 | Yes |
| 9 | 103230396 | UCHL5 |  |  | 4069 | 1.518 | 0.0716 | Yes |
| 10 | 103236978 | ELOC |  |  | 4135 | 1.510 | 0.0919 | Yes |
| 11 | 103219057 | RAD23B |  |  | 4195 | 1.502 | 0.1125 | Yes |
| 12 | 103240980 | PSMB1 |  |  | 4329 | 1.481 | 0.1294 | Yes |
| 13 | 103216597 | CACUL1 |  |  | 4363 | 1.477 | 0.1507 | Yes |
| 14 | 103239807 | PSMA1 |  |  | 4529 | 1.456 | 0.1657 | Yes |
| 15 | 103238047 | LOC103238047 |  |  | 4673 | 1.439 | 0.1815 | Yes |
| 16 | 103227180 | CUL1 |  |  | 4689 | 1.437 | 0.2030 | Yes |
| 17 | 103232443 | SIAH1 |  |  | 4733 | 1.432 | 0.2232 | Yes |
| 18 | 103239889 | PSMB7 |  |  | 5265 | 1.373 | 0.2206 | Yes |
| 19 | 103238109 | CUL2 |  |  | 5413 | 1.358 | 0.2349 | Yes |
| 20 | 103241778 | NGLY1 |  |  | 5515 | 1.347 | 0.2512 | Yes |
| 21 | 103214836 | CUL4A |  |  | 5547 | 1.343 | 0.2705 | Yes |
| 22 | 103243135 | PSMA7 |  |  | 5688 | 1.332 | 0.2848 | Yes |
| 23 | 103244544 | SKP1 |  |  | 5829 | 1.319 | 0.2989 | Yes |
| 24 | 103248397 | CUL5 |  |  | 5879 | 1.313 | 0.3169 | Yes |
| 25 | 103229087 | PSMA3 |  |  | 6735 | 1.240 | 0.2978 | Yes |
| 26 | 103225984 | UBE4B |  |  | 7217 | 1.204 | 0.2948 | Yes |
| 27 | 103246532 | LOC103246532 |  |  | 7257 | 1.200 | 0.3116 | Yes |
| 28 | 103223000 | UFD1 |  |  | 7764 | 1.160 | 0.3068 | Yes |
| 29 | 103242203 | PSMB6 |  |  | 7964 | 1.146 | 0.3156 | Yes |
| 30 | 103218996 | CDC26 |  |  | 8044 | 1.139 | 0.3296 | Yes |
| 31 | 103231459 | PSMB11 |  |  | 8121 | 1.134 | 0.3437 | Yes |
| 32 | 103234004 | RAD23A |  |  | 9084 | 1.080 | 0.3173 | Yes |
| 33 | 103224487 | LOC103224487 |  |  | 9107 | 1.079 | 0.3330 | Yes |
| 34 | 103243561 | PSMB3 |  |  | 9126 | 1.077 | 0.3488 | Yes |
| 35 | 103246177 | UCHL1 |  |  | 9200 | 1.072 | 0.3620 | Yes |
| 36 | 103227728 | BAP1 |  |  | 9377 | 1.061 | 0.3705 | Yes |
| 37 | 103247359 | CUL4B |  |  | 9625 | 1.046 | 0.3756 | Yes |
| 38 | 103245751 | UBR1 |  |  | 9689 | 1.042 | 0.3889 | Yes |
| 39 | 103245250 | PSMA4 |  |  | 9695 | 1.041 | 0.4047 | Yes |
| 40 | 103231457 | PSMB5 |  |  | 9933 | 1.025 | 0.4099 | Yes |
| 41 | 103224033 | PSMB4 |  |  | 10344 | 1.001 | 0.4070 | Yes |
| 42 | 103221472 | CUL9 |  |  | 10375 | 0.999 | 0.4210 | Yes |
| 43 | 103248589 | UBE4A |  |  | 10626 | 0.982 | 0.4250 | Yes |
| 44 | 103228819 | PSMA6 |  |  | 10943 | 0.960 | 0.4257 | Yes |
| 45 | 103225129 | PSMB2 |  |  | 12475 | 0.864 | 0.3705 | No |
| 46 | 103243526 | PSMD3 |  |  | 12558 | 0.859 | 0.3801 | No |
| 47 | 103239578 | ANAPC2 |  |  | 13127 | 0.825 | 0.3674 | No |
| 48 | 103221475 | CUL7 |  |  | 13465 | 0.803 | 0.3647 | No |
| 49 | 103238477 | LOC103238477 |  |  | 17245 | 0.554 | 0.2041 | No |
| 50 | 103221690 | PSMB8 |  |  | 18129 | 0.478 | 0.1720 | No |
| 51 | 103233253 | PSMB10 |  |  | 19266 | 0.364 | 0.1268 | No |
| 52 | 103221693 | PSMB9 |  |  | 19351 | 0.356 | 0.1285 | No |
| 53 | 103241738 | LOC103241738 |  |  | 19595 | 0.329 | 0.1227 | No |
| 54 | 103246089 | LOC103246089 |  |  | 20527 | 0.185 | 0.0839 | No |
Table: GSEA details
[plain text format]

  

Fig 2: PROTEIN\_CATABOLIC\_PROCESS(GO:0030163)
  
Blue-Pink O' Gram in the Space of the Analyzed GeneSet

  

Fig 3: PROTEIN\_CATABOLIC\_PROCESS(GO:0030163): Random ES distribution
  
Gene set null distribution of ES for
 **PROTEIN\_CATABOLIC\_PROCESS(GO:0030163)**
